# (*R*)-(−)-Quinuclidin-3-ol

**DOI:** 10.1107/S1600536813026998

**Published:** 2013-10-19

**Authors:** Yoann Rousselin, Alexandre Clavel, Isabelle Bonnaventure

**Affiliations:** a Institut de Chimie Moleculaire de l’Universite de Bourgogne - ICMUB, UMR CNRS 6302, Universite de Bourgogne, 9, Av. Alain Savary, 21078 Dijon Cedex, France; b CordenPharma – Synkem, 47 rue de Longvic, 21301 Chenove, France

## Abstract

The structure of the title compound [alternatively called (*R*)-(−)-1-aza­bicyclo­[2.2.2]octan-3-ol], C_7_H_13_NO, at 100 K has hexa­gonal (*P*6_1_) symmetry. The structure shows a twist along the C—N pseudo-threefold axis. In the crystal, mol­ecules are linked *via* O—H⋯N hydrogen bonds, forming infinite chains along the *c-*axis direction. The crystal studied was twinned by merohedry (twin law: 010, 100, 00-1; population: 0.925:0.075)

## Related literature

The title compound is a key building block for the syntheses of muscarinic receptor ligands, including solifenacin (Naito *et al.*, 2005 ▶), revatropate (Alabaster, 1997 ▶) and talsaclidine (Leusch *et al.*, 2000 ▶). For properties of the title compound, see: Bosak *et al.* (2005 ▶); Carroll *et al.* (1991 ▶); Frackenpohl & Hoffmann (2000 ▶); Day & Motherwell (2006 ▶); Malone & Armstrong (2006 ▶); Siczek & Lis (2008 ▶); Sterling *et al.* (1988 ▶). For puckering parameters, see: Cremer & Pople (1975 ▶); For absolute configuration, see: Flack (1983 ▶); The twin law was determined using *TwinRotMat* implemented in *PLATON* (Spek, 2009 ▶).
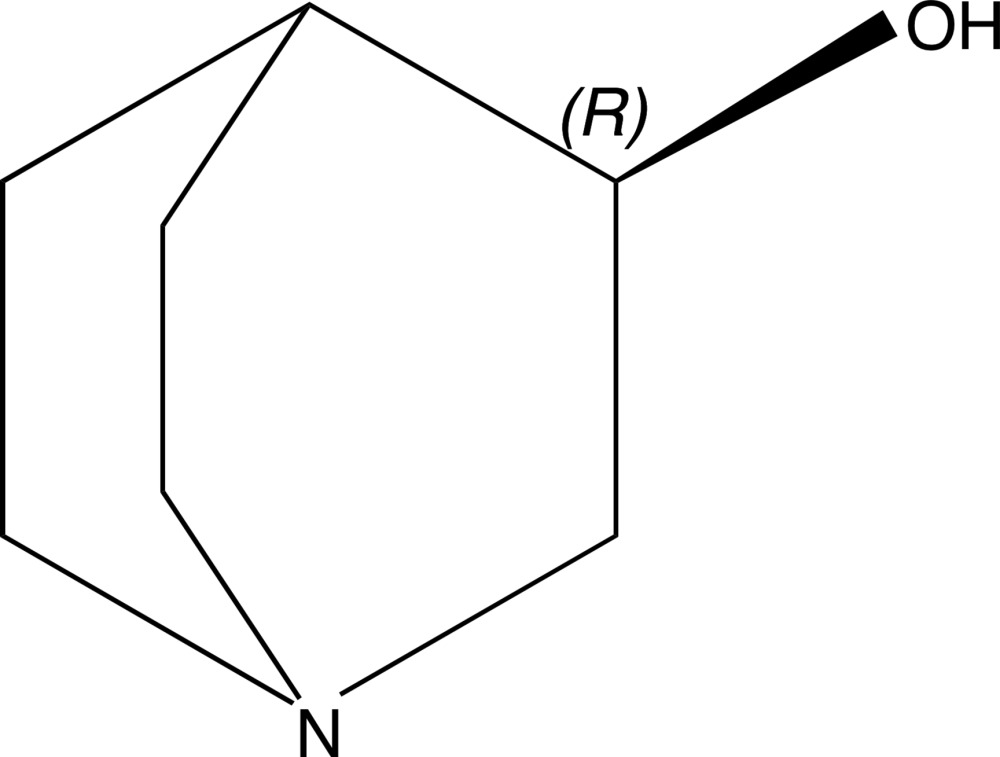


## Experimental

### 

#### Crystal data

C_7_H_13_NO
*M* *_r_* = 127.18
Hexagonal, 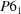

*a* = 6.2076 (3) Å
*c* = 29.8731 (13) Å
*V* = 996.91 (11) Å^3^
*Z* = 6
Cu *K*α_1_ radiation
μ = 0.67 mm^−1^
*T* = 100 K
0.58 × 0.44 × 0.32 mm

#### Data collection

Bruker D8 VENTURE diffractometer
Absorption correction: numerical (*SADABS*; Bruker, 2012 ▶) *T*_min_ = 0.58, *T*_max_ = 0.74
15447 measured reflections
1240 independent reflections
1240 reflections with *I* > 2σ(*I*)
*R*_int_ = 0.026

#### Refinement

*R*[*F* ^2^ > 2σ(*F* ^2^)] = 0.023
*wR*(*F* ^2^) = 0.064
*S* = 1.15
1240 reflections
85 parameters
1 restraint
H-atom parameters constrained
Δρ_max_ = 0.23 e Å^−3^
Δρ_min_ = −0.12 e Å^−3^
Absolute structure: Parsons & Flack (2004 ▶).
Absolute structure parameter: 0.01 (4)

### 

Data collection: *APEX2* (Bruker, 2012 ▶); cell refinement: *SAINT* (Bruker, 2012 ▶); data reduction: *SAINT*; program(s) used to solve structure: *SHELXS97* (Sheldrick, 2008 ▶); program(s) used to refine structure: *SHELXL97* (Sheldrick, 2008 ▶); molecular graphics: *OLEX2* (Dolomanov *et al.*, 2009 ▶); software used to prepare material for publication: *OLEX2*.

## Supplementary Material

Crystal structure: contains datablock(s) I. DOI: 10.1107/S1600536813026998/bg2517sup1.cif

Structure factors: contains datablock(s) I. DOI: 10.1107/S1600536813026998/bg2517Isup2.hkl

Supplementary material file. DOI: 10.1107/S1600536813026998/bg2517Isup3.cml

Additional supplementary materials:  crystallographic information; 3D view; checkCIF report

## Figures and Tables

**Table 1:** Hydrogen-bond geometry (Å, °)

*D*—H⋯*A*	*D*—H	H⋯*A*	*D*⋯*A*	*D*—H⋯*A*
O1—H1⋯N1^i^	0.84	2.00	2.8366 (19)	176

Symmetry code: (i) 
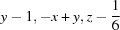
.

**Fig. 1.**
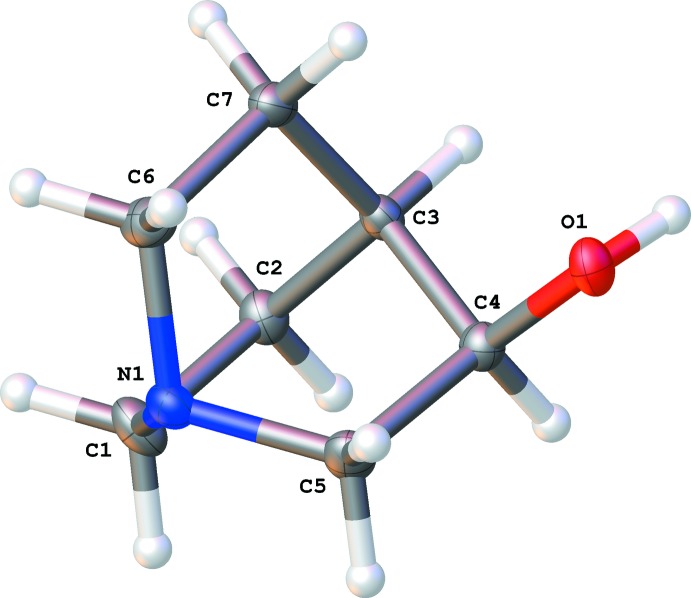
A view of (R)-(-)-quinuclidin-3-ol with atom labelling scheme. The thermal displacement ellipsoids are drawn at 50% probability level.

**Fig. 2.**
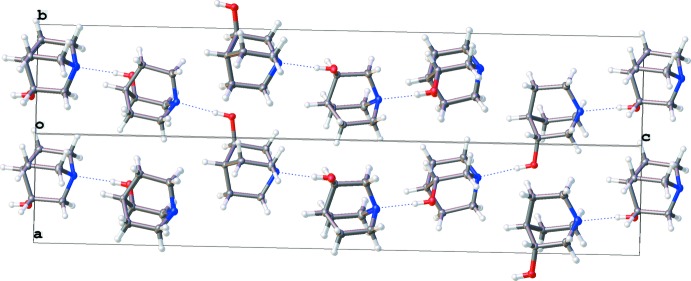
A view of molecular packing showing chains running along the c direction. The hydrogen bonds are shown as dashed lines. The thermal displacement ellipsoids are drawn at 50% probability level.

## supplementary crystallographic information

### 1. Comment

(R)-(-)-quinuclidin-3-ol (Figure 1) is a key building block for the syntheses of muscarinic receptor ligands, including solifenacin (M3 receptor antagonist),(Naito *et al.*, 2005) revatropate (M3 receptor antagonist),(Alabaster, 1997) and talsaclidine (M1 receptor agonist),(Leusch *et al.*, 2000).

The asymetric unit of the crystal (Figure 1) consists of one single (R)-(-)-quinuclidin-3-ol molecule.

The quinuclidinol moiety has pseudo-threefold symmetry about the N1-C3 axis with C6-N1-C1, C1-N1-C5 and C5-N1-N6 angles of 108.86 (15)°, 108.73 (14)° and 108.72 (14)° respectively, and N1-C1-C2-C3, N1-C6-C7-C3 and N1-C5-C4-C3 torsion angles of -0.9 (3)°, 0.4 (2)° and 0.2 (2)° respectively.

The three piperidine rings formed by (N1, C1, C2, C3, C4, C5), (N1, C5, C4, C3, C7, C6) and (N1, C6, C7, C3, C2, C1) adopt a boat conformation with total puckering amplitutdes QT of 0.8218 (0) (with Θ = 91.84 (0)° and φ = -0.2 (0)°), QT of 0.8141 (1) (with Θ = 91.55 (0)° and φ = 0.19 (0)°) and QT of 0.8123 (0) (with Θ = 90.95 (0)° and φ = -0.17 (0)°), respectively (Cremer & Pople, (1975)).

There is a hydrogen bond (Table 1) which links molecules into infinite chains along the c axis (Figure 2).

### 2. Refinement

All H atoms, on carbon atom or oxygen atom, were placed at calculated positions using a riding model with C-H = 1 Å (methine), 0.99 Å (methylene) or O-H = 0.84 Å with Uiso(H) = 1.2Ueq(CH), Uiso(H) = 1.2Ueq(CH_2_) or Uiso(H) = 1.5Ueq(OH).

TWIN/BASF refinement type was used to determine absolute configuration from anomalous scattering using the Flack method. (Flack, 1983). The structure display a merohedral twinning and the twin law was found by using TwinRotMat implemented in Platon (Spek, 2009). The use of twin law (0 1 0 1 0 0 0 0 -1) with a population of 0.925/0.075 reduced the R1(for I> 2σ(I)) and Flack parameter from 4.96%, 0.2 (9) to 2.34%, 0.01 (4).

### Crystal data

**Table:**

C_7_H_13_NO	*D*_x_ = 1.271 Mg m^−^^3^
*M**_r_* = 127.18	Melting point: 492(2) K
Hexagonal, *P*6_1_	Cu *K*α_1_ radiation, λ = 1.54178 Å
*a* = 6.2076 (3) Å	µ = 0.67 mm^−^^1^
*c* = 29.8731 (13) Å	*T* = 100 K
*V* = 996.91 (11) Å^3^	Prism, clear light colourless
*Z* = 6	0.58 × 0.44 × 0.32 mm
*F*(000) = 420	

### Data collection

**Table:**

Bruker D8 VENTURE diffractometer	1240 independent reflections
Radiation source: sealed X-ray tube, high brilliance microfocus sealed tube, Cu	1240 reflections with *I* > 2σ(*I*)
Graphite monochromator	*R*_int_ = 0.026
Detector resolution: 1024 x 1024 pixels mm^-1^	θ_max_ = 69.2°, θ_min_ = 4.4°
φ and ω scans	*h* = −7→7
Absorption correction: numerical (*SADABS*; Bruker, 2012)	*k* = −7→7
*T*_min_ = 0.58, *T*_max_ = 0.74	*l* = −34→35
15447 measured reflections	

### Refinement

**Table:**

Refinement on *F*^2^	Hydrogen site location: inferred from neighbouring sites
Least-squares matrix: full	H-atom parameters constrained
*R*[*F*^2^ > 2σ(*F*^2^)] = 0.023	*w* = 1/[σ^2^(*F*_o_^2^) + (0.0394*P*)^2^ + 0.1183*P*] where *P* = (*F*_o_^2^ + 2*F*_c_^2^)/3
*wR*(*F*^2^) = 0.064	(Δ/σ)_max_ < 0.001
*S* = 1.15	Δρ_max_ = 0.23 e Å^−^^3^
1240 reflections	Δρ_min_ = −0.12 e Å^−^^3^
85 parameters	Extinction correction: *SHELXL97* (Sheldrick, 2008), Fc^*^=kFc[1+0.001xFc^2^λ^3^/sin(2θ)]^-1/4^
1 restraint	Extinction coefficient: 0.0156 (15)
0 constraints	Absolute structure: Parsons & Flack (2004).
Primary atom site location: structure-invariant direct methods	Absolute structure parameter: 0.01 (4)

### Special details

**Table:**

Geometry. All e.s.d.'s (except the e.s.d. in the dihedral angle between two l.s. planes) are estimated using the full covariance matrix. The cell e.s.d.'s are taken into account individually in the estimation of e.s.d.'s in distances, angles and torsion angles; correlations between e.s.d.'s in cell parameters are only used when they are defined by crystal symmetry. An approximate (isotropic) treatment of cell e.s.d.'s is used for estimating e.s.d.'s involving l.s. planes.
Refinement. Refined as a 2-component twin.

### Fractional atomic coordinates and isotropic or equivalent isotropic displacement parameters (Å^2^)

**Table:**

	*x*	*y*	*z*	*U*_iso_*/*U*_eq_	
O1	−0.1928 (2)	0.4528 (2)	0.48963 (4)	0.0175 (3)	
H1	−0.2253	0.4264	0.4622	0.026*	
N1	0.3057 (3)	0.6832 (3)	0.56404 (5)	0.0157 (4)	
C2	0.5095 (3)	0.8018 (3)	0.48879 (6)	0.0166 (4)	
H2A	0.5325	0.9500	0.4722	0.020*	
H2B	0.6378	0.7625	0.4786	0.020*	
C4	0.0565 (3)	0.6495 (3)	0.49520 (5)	0.0149 (4)	
H4	0.0812	0.7999	0.4785	0.018*	
C1	0.5354 (4)	0.8550 (4)	0.53973 (6)	0.0208 (4)	
H1A	0.6752	0.8380	0.5515	0.025*	
H1B	0.5744	1.0283	0.5450	0.025*	
C7	0.2139 (3)	0.3542 (3)	0.50634 (5)	0.0159 (4)	
H7A	0.3369	0.3067	0.4966	0.019*	
H7B	0.0450	0.2111	0.5013	0.019*	
C5	0.0986 (4)	0.7091 (4)	0.54597 (5)	0.0188 (4)	
H5A	0.1340	0.8814	0.5510	0.023*	
H5B	−0.0557	0.5956	0.5624	0.023*	
C3	0.2487 (3)	0.5801 (3)	0.47977 (6)	0.0141 (4)	
H3	0.2279	0.5406	0.4471	0.017*	
C6	0.2509 (4)	0.4253 (4)	0.55649 (6)	0.0204 (4)	
H6A	0.0984	0.3095	0.5732	0.024*	
H6B	0.3897	0.4068	0.5684	0.024*	

### Atomic displacement parameters (Å^2^)

**Table:**

	*U*^11^	*U*^22^	*U*^33^	*U*^12^	*U*^13^	*U*^23^
O1	0.0134 (6)	0.0222 (6)	0.0150 (6)	0.0076 (5)	−0.0012 (4)	−0.0004 (5)
N1	0.0176 (7)	0.0155 (8)	0.0128 (7)	0.0074 (6)	−0.0005 (6)	−0.0014 (5)
C2	0.0149 (8)	0.0186 (9)	0.0145 (8)	0.0069 (7)	0.0009 (7)	0.0010 (6)
C4	0.0150 (8)	0.0161 (8)	0.0138 (8)	0.0079 (7)	−0.0002 (6)	0.0006 (7)
C1	0.0176 (9)	0.0215 (9)	0.0164 (9)	0.0045 (8)	−0.0020 (6)	−0.0034 (7)
C7	0.0150 (8)	0.0151 (8)	0.0177 (8)	0.0077 (7)	−0.0012 (6)	−0.0028 (7)
C5	0.0207 (9)	0.0237 (9)	0.0153 (8)	0.0135 (8)	−0.0006 (7)	−0.0042 (7)
C3	0.0135 (8)	0.0158 (8)	0.0124 (8)	0.0068 (7)	−0.0011 (6)	−0.0024 (7)
C6	0.0292 (9)	0.0186 (9)	0.0159 (9)	0.0139 (8)	−0.0003 (8)	0.0013 (7)

### Geometric parameters (Å, º)

**Table:**

O1—H1	0.8400	C1—H1A	0.9900
O1—C4	1.423 (2)	C1—H1B	0.9900
N1—C1	1.475 (2)	C7—H7A	0.9900
N1—C5	1.475 (2)	C7—H7B	0.9900
N1—C6	1.479 (2)	C7—C3	1.530 (2)
C2—H2A	0.9900	C7—C6	1.546 (2)
C2—H2B	0.9900	C5—H5A	0.9900
C2—C1	1.548 (2)	C5—H5B	0.9900
C2—C3	1.536 (2)	C3—H3	1.0000
C4—H4	1.0000	C6—H6A	0.9900
C4—C5	1.552 (2)	C6—H6B	0.9900
C4—C3	1.528 (2)		
			
C4—O1—H1	109.5	C3—C7—H7A	110.1
C1—N1—C6	108.86 (15)	C3—C7—H7B	110.1
C5—N1—C1	108.73 (14)	C3—C7—C6	107.96 (13)
C5—N1—C6	108.72 (14)	C6—C7—H7A	110.1
H2A—C2—H2B	108.4	C6—C7—H7B	110.1
C1—C2—H2A	110.0	N1—C5—C4	112.56 (14)
C1—C2—H2B	110.0	N1—C5—H5A	109.1
C3—C2—H2A	110.0	N1—C5—H5B	109.1
C3—C2—H2B	110.0	C4—C5—H5A	109.1
C3—C2—C1	108.29 (15)	C4—C5—H5B	109.1
O1—C4—H4	109.6	H5A—C5—H5B	107.8
O1—C4—C5	107.46 (13)	C2—C3—H3	109.9
O1—C4—C3	112.96 (14)	C4—C3—C2	108.41 (14)
C5—C4—H4	109.6	C4—C3—C7	109.25 (14)
C3—C4—H4	109.6	C4—C3—H3	109.9
C3—C4—C5	107.45 (13)	C7—C3—C2	109.45 (14)
N1—C1—C2	111.72 (14)	C7—C3—H3	109.9
N1—C1—H1A	109.3	N1—C6—C7	112.19 (14)
N1—C1—H1B	109.3	N1—C6—H6A	109.2
C2—C1—H1A	109.3	N1—C6—H6B	109.2
C2—C1—H1B	109.3	C7—C6—H6A	109.2
H1A—C1—H1B	107.9	C7—C6—H6B	109.2
H7A—C7—H7B	108.4	H6A—C6—H6B	107.9
			
O1—C4—C5—N1	122.04 (17)	C5—C4—C3—C2	−59.75 (17)
O1—C4—C3—C2	−178.09 (13)	C5—C4—C3—C7	59.46 (17)
O1—C4—C3—C7	−58.88 (17)	C3—C2—C1—N1	−0.9 (2)
C1—N1—C5—C4	59.38 (18)	C3—C4—C5—N1	0.20 (19)
C1—N1—C6—C7	−59.78 (19)	C3—C7—C6—N1	0.4 (2)
C1—C2—C3—C4	60.54 (19)	C6—N1—C1—C2	59.72 (19)
C1—C2—C3—C7	−58.55 (18)	C6—N1—C5—C4	−59.01 (19)
C5—N1—C1—C2	−58.6 (2)	C6—C7—C3—C2	58.56 (18)
C5—N1—C6—C7	58.53 (19)	C6—C7—C3—C4	−60.01 (17)

### Hydrogen-bond geometry (Å, º)

**Table:**

*D*—H···*A*	*D*—H	H···*A*	*D*···*A*	*D*—H···*A*
O1—H1···N1^i^	0.84	2.00	2.8366 (19)	176

Symmetry code: (i) *y*−1, −*x*+*y*, *z*−1/6.
